# Suppression of Short-Channel Effects in AlGaN/GaN HEMTs Using SiN_x_ Stress-Engineered Technique

**DOI:** 10.3390/nano14221817

**Published:** 2024-11-13

**Authors:** Chenkai Deng, Chuying Tang, Peiran Wang, Wei-Chih Cheng, Fangzhou Du, Kangyao Wen, Yi Zhang, Yang Jiang, Nick Tao, Qing Wang, Hongyu Yu

**Affiliations:** 1 School of Electronic Information and Engineering, Harbin Institute of Technology, Harbin 150001, China; 12149033@mail.sustech.edu.cn (C.D.); 12049024@mail.sustech.edu.cn (C.T.); 2 School of Microelectronics, Southern University of Science and Technology, Shenzhen 518055, China; 12333281@mail.sustech.edu.cn (P.W.); 11811803@mail.sustech.edu.cn (F.D.); 3 State Key Laboratory of ASIC and System, School of Microelectronics, Fudan University, Shanghai 200433, China; 22112020122@m.fudan.edu.cn; 4 Faculty of Engineering, The University of Hong Kong, Hong Kong 999077, China; zhangyi97@connect.hku.hk (Y.Z.); 11510044@mail.sustech.edu.cn (Y.J.); 5 Maxscend Microelectronics Co., Ltd., Wuxi 214072, China; nick.tao@maxscend.com; 6 Engineering Research Center of Integrated Circuits for Next-Generation Communications Ministry of Education, Southern University of Science and Technology, Shenzhen 518055, China

**Keywords:** GaN HEMTs, SCEs, DIBL, SiN_x_ stress engineered

## Abstract

In this work, we present the novel application of SiN_x_ stress-engineering techniques for the suppression of short-channel effects in AlGaN/GaN high-electron-mobility transistors (HEMTs), accompanied by a comprehensive analysis of the underlying mechanisms. The compressive stress SiN_x_ passivation significantly enhances the barrier height at the heterojunction beneath the gate, maintaining it above the quasi-Fermi level even as *V_ds_* rises to 20 V. As a result, in GaN devices with a gate length of 160 nm, the devices with compressive stress SiN_x_ passivation exhibit significantly lower drain-induced barrier lowering (DIBL) factors of 2.25 mV/V, 2.56 mV/V, 4.71 mV/V, and 3.84 mV/V corresponding to drain bias voltages of 5 V, 10 V, 15 V, and 20 V, respectively. Furthermore, as *V_ds_* increases, there is an insignificant degradation in transconductance, subthreshold swing, leakage current, or output conductance. In contrast, the devices with stress-free passivation show relatively higher DIBL factors (greater than 20 mV/V) and substantial degradation in pinch-off performance and output characteristics. These results demonstrate that the SiN_x_ stress-engineering technique is an attractive technique to facilitate high-performance and high-reliability GaN-based HEMTs for radio frequency (RF) electronics applications.

## 1. Introduction

Gallium nitride (GaN) has garnered significant attention for radio frequency (RF) power applications, owing to its exceptional material properties, including a high saturated electron velocity, high critical breakdown field, and high-temperature stability [1,2,3]. When operating at higher frequencies, ranging from the Ka to the W band, a reduction in the short gate is indispensable [4,5,6]. However, as the gate length decreases, the aspect ratio (gate length (*L*_g_)/barrier thickness (*T*_barrier_)) worsens [7]. This deterioration weakens the gate’s control over channel electrons, causing the drain bias to affect the entire potential barrier in the channel. This reduction in control allows the drain bias to influence the entire potential barrier across the channel, leading to short-channel effects (SCEs) [8,9,10].

**Figure 1:**
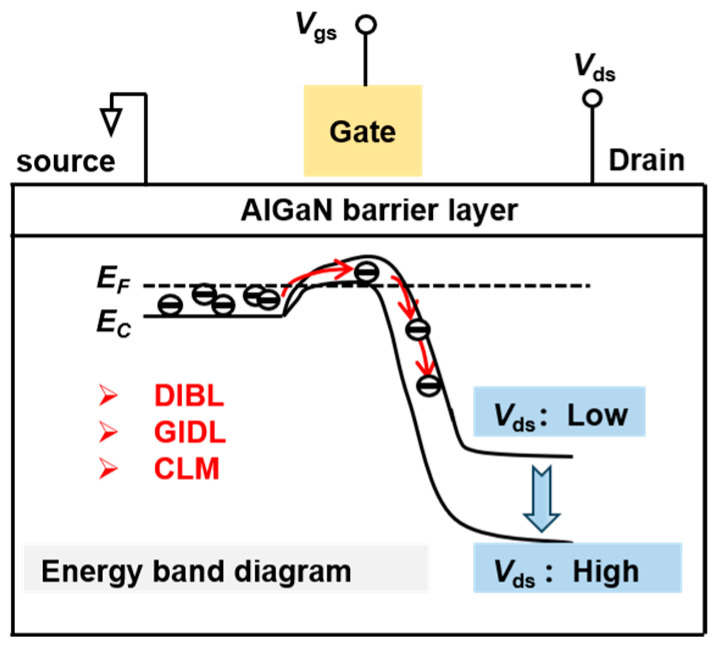
Schematic diagram of SCEs for GaN HEMTs.

Figure 1 shows a schematic diagram of the SCEs in GaN N-polar high-electron-mobility transistors (HEMTs). As the drain bias increases, the potential barrier between the source and drain decreases, eventually falling below the Fermi level, severely impacting device performance. Drain-induced barrier lowering (DIBL) is a significant SCE observed in short-gate-length devices [11]. When a drain bias is applied, the barrier height beneath the gate decreases, leading to an increased injection of carriers into the channel, which leads to a negative shift in threshold voltage (*V_th_*) [12,13,14]. In addition, the high electric field under the gate causes significant band bending, facilitating electron tunneling in the drain region and increasing the off-state leakage current, known as gate-induced drain leakage (GIDL) [15,16,17]. Another notable SCE is channel length modulation (CLM) [18], which is clearly observable in the drain output characteristic curve of short gate devices. The strong electric field causes velocity saturation to occur earlier at the drain side, effectively reducing the effective channel length. Consequently, this leads to an increase in drain current with rising drain voltage, even within the saturation region [19,20].

To date, various solutions have been proposed to suppress SCEs and improve device performance. An ultra-thin barrier layer structure can increase the aspect ratio to suppress SCEs [21], but thinning of the barrier layer reduces the concentration of the two-dimensional electron gas (2DEG), negatively affecting the device’s output characteristics. Due to strong piezoelectric polarization effects, InAlN/GaN [22,23] and AlN/GaN [24,25] heterostructures can maintain a high concentration of 2DEG even with a thin barrier layer. N-polar HEMTs exhibit superior SCE suppression characteristics because their inverted HEMT structure maintains rigorous channel confinement [12]. However, growing high-quality InAlN/GaN, ultra-thin AlN/GaN, and N-polar AlGaN/GaN heterojunctions is challenging. Gate recess technology effectively enhances gate control by decreasing the gate-to-channel distance, thus improving the aspect ratio and mitigating SCEs [26,27]. However, while this approach can significantly enhance on-state performance, it often leads to increased gate leakage and reduced breakdown voltage, potentially compromising device reliability. Fin HEMT structures improve electrostatic control of the channel by increasing the effective channel width [25,28]. However, the fabrication of fin structures is complex and necessitates advanced photolithography techniques, which complicate the manufacturing process and may lead to higher production costs. Similarly, dual metal gate designs can reduce SCEs by employing different metal materials to optimize work functions, thereby enhancing overall gate control [29,30]. Although it has been shown to improve device performance, its complex fabrication process makes it less practical for mass production.

In this work, we propose the SiN_x_ stress-engineered technique as a novel straightforward method to suppress SCEs while simultaneously enhancing pinch-off and output characteristics. By using compressive stress SiN_x_ passivation, devices with relatively poor aspect ratios demonstrate significant suppression of SCEs, achieving a DIBL factor as low as 3.84 mV/V at *V_ds_* = 20 V. Moreover, these devices exhibit improvements in leakage current, transconductance, and subthreshold swing. With increasing *V_ds_* bias, there is negligible degradation in pinch-off and output characteristics, highlighting the potential of the SiN_x_ stress-engineered technique as an effective solution for mitigating SCEs.

## 2. Device Structure and Fabrication Process

**Figure 2:**
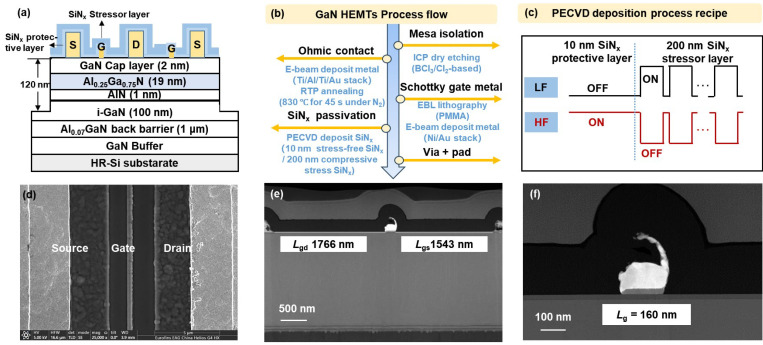
(**a**) Schematic diagram and (**b**) process flow of AlGaN/GaN-on-Si RF devices. (**c**) Process recipe of SiN_x_ stressor passivation by PECVD. (**d**) SEM images of overall device. TEM images of (**e**) overall GaN HEMT and (**f**) gate region.

The epitaxial structure, shown in Figure 2a, comprises a 1.05 μm buffer layer, a 1 μm Al_0.07_GaN back-barrier layer, a 100 nm i-GaN channel layer, a 1 nm AlN spacer, a 19 nm Al_0.25_Ga_0.75_N barrier layer, and a 2 nm GaN cap layer. This back-barrier HEMT structure maintains rigorous channel confinement, thus suppressing SCEs [31].

As shown in Figure 2b, the device fabrication process begins with device isolation using BCl_3_/Cl_2_-based inductively coupled plasma dry etching. This is followed by the deposition of a Ti/Al/Ti/Au metal stack, which is then annealed at 830 °C for 45 s in ambient nitrogen to form the source and drain ohmic contacts. The gate region is subsequently patterned using electron beam lithography (EBL) with polymethyl methacrylate (PMMA), and the Ni/Au metal gate is fabricated using an e-beam evaporator. Subsequently, a compressively stressed SiN_x_ passivation layer is applied using plasma-enhanced chemical vapor deposition (PECVD), followed by a CHF_3_-based via opening etching process. Finally, Ti/Au (20/180 nm) metal pads are deposited. Figure 2d–f shows the SEM images of the overall device and the TEM images of the gate region of the fabricated GaN RF device, along with the measured device dimensions. The reported devices feature a *L*_g_ of 0.16 μm, a gate width (*W*_g_) of 2 × 25 μm, a gate drain spacing (*L*_gd_) of 1.766 μm, and a gate source spacing (*L*_gs_) of 1.543 μm.

In this study, the compressive stress SiN_x_ passivation layer was deposited using an Oxford PlasmaPro80 PECVD system. The process utilized a SiH_4_ gas flow rate of 6.5 sccm, NH_3_ at 4.0 sccm, and a N_2_ carrier gas at 100 sccm, consistent with the equipment’s standard settings. The PECVD parameters—such as temperature, pressure, gas chemistry, power density, and the frequency of the RF excitation source—significantly influence the stress of the deposited film. High-energy ion bombardment resulting from low-frequency additions (below 1 MHz) promotes film densification, causing the film to expand relative to its volume and thereby contributing to the formation of compressive stress within the deposited film [32]. Consequently, adjusting the duty cycles of high and low frequencies (HFs and LFs) in the PECVD reactor effectively regulates the stress in the thin films. In this setup, the HF power is set to 20 W, while the LF power is maintained at 100 W. However, high-energy ion bombardment can also damage the AlGaN surface and introduce defects. To mitigate this, a 10 nm full-HF SiN_x_ protective layer is deposited as the first passivation layer to reduce surface damage. The stress level in the SiN_x_ passivation layer is critical for GaN devices, as excessive stress can result in poor film quality, reliability issues, and reduced mobility, while insufficient stress may fail to deliver the desired enhancements in device performance. Thus, we optimized the PECVD process to deposit a 200 nm compressive stress SiN_x_ passivation layer with a LF duty cycle of 95% and a 200 nm stress-free SiN_x_ passivation layer with a duty cycle of 45%, resulting in significant improvements in device performance.

## 3. Results and Discussion

### 3.1. DC Characterization

**Figure 3:**
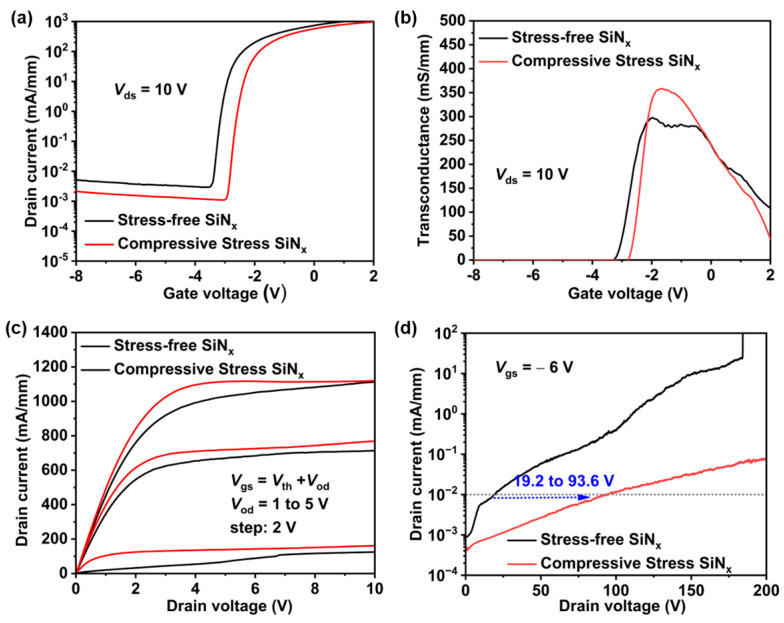
DC characteristics of devices with stress-free SiN_x_ and compressive SiN_x_ passivation. (**a**) Transfer characteristics and (**b**) transconductance under *V_ds_* = 10 V. (**c**) Output characteristics at *V*_od_ = 1 to 5 V. (**d**) Breakdown voltage under *V_gs_* = −6 V.

DC measurements were conducted using a Keithley 4200 semiconductor parameter analyzer. As shown in Figure 2a, the compressive stress SiN_x_ passivation mitigates polarization effects in AlGaN/GaN HEMTs, resulting in a positive shift in *V_th_*. It also reduces the polarization electric field, effectively suppressing Fowler–Nordheim (FN) tunneling and lowering the leakage current [33]. As illustrated in Figure 3b, the introduction of compressive stress SiN_x_ passivation has significantly improved the device’s breakdown voltage, enhancing reliability under high-voltage operation. Figure 3c,d also show clear improvements in peak transconductance and saturated output current, consistent with trends observed in our previous studies [34]. This indicates a positive impact of SiN_x_ stress engineering on overall DC performance.

### 3.2. Short-Channel Effect Results

**Figure 4:**
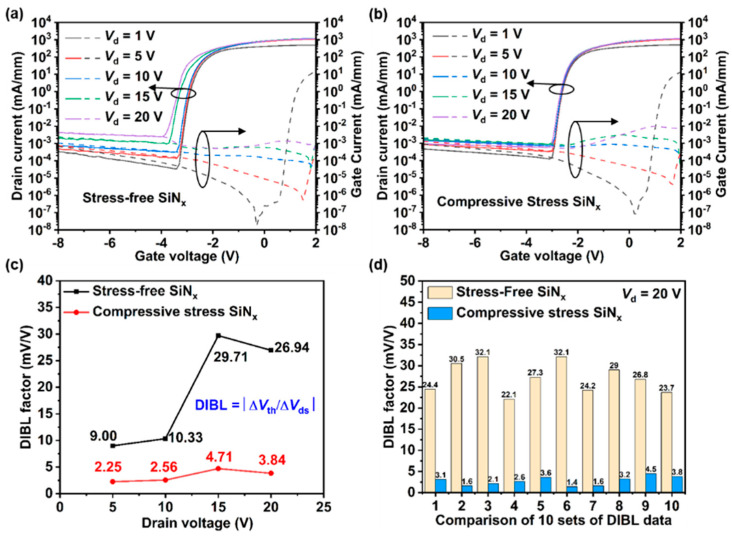
The transfer characteristics under *V_ds_* = 1 to 20 V of devices with (**a**) stress-free SiN_x_ passivation and (**b**) compressive stress SiN_x_ passivation. (**c**) The DIBL factor for two types of devices under various *V_ds_* biases. (**d**) The DIBL for 15 devices of compressive stress SiN_x_ and stress-free SiN_x_ passivation at a bias of *V_ds_* = 20 V.

SCEs can significantly impact GaN RF devices, especially when the gate size is very small, leading to shifts in *V_th_*, degradation of pinch-off performance, and increased output conductance, ultimately reducing performance and efficiency in both off-state and on-state operations. To explore the effects of the SiN_x_ stress-engineered technique on SCEs, we investigated the CLM, DIBL, and GIDL phenomena in GaN HEMTs with compressive stress passivation and stress-free passivation, as shown in Figure 3c and Figure 4. As shown in Figure 3c, when *V*_od_ = 1 V, the drain current of devices with stress-free SiN_x_ passivation gradually increases with increasing *V_ds_*, saturating only at 7 V. In contrast, the output current of devices with compressive stress SiN_x_ passivation saturates before reaching 2 V, strongly confirming the effectiveness of stress-engineering techniques in reducing output conductance and suppressing the CLM effects. Figure 4a,b display the transfer characteristics of each GaN HEMT under *V_ds_* = 1 V, 5 V, 10 V, 15 V, and 20 V, respectively. As the *V_ds_* bias increases, devices with stress-free passivation exhibit a more pronounced negative shift (0.5 V) in *V_th_* and an increase in leakage current (more than an order of magnitude). Conversely, devices with compressive stress SiN_x_ passivation show negligible performance degradation. The DIBL factor is a valuable metric for evaluating SCE suppression. To more accurately evaluate the impact of SiN_x_ stress engineering on SCE suppression, we calculated the DIBL factors for both types of devices under various *V_ds_* biases, as illustrated in Figure 4c. The formula for calculating the DIBL factor is as follows:

(1) $$DIBL=|\Delta V_{th}/\Delta V_{ds}|$$

Due to the adoption of a back-barrier structure in the epitaxial material, devices with stress-free SiN_x_ passivation exhibit relatively low DIBL factors (less than 10 mV/V) at *V_ds_* = 5 V and 10 V. However, as the *V_ds_* bias increases to 20 V, a more pronounced DIBL phenomenon becomes apparent in these devices (approaching 30 mV/V). Significantly, devices with compressive stress SiN_x_ passivation maintain an excellent suppression of DIBL (less than 5 mV/V) with increasing *V_ds_* bias. Figure 4d illustrates the DIBL for 10 devices with compressive stress SiN_x_ and stress-free SiN_x_ passivation at a bias of *V_ds_* = 20 V. Devices with stress-free SiN_x_ passivation show DIBL factors mostly ranging from 20 to 30 mV/V, with some exceeding 30 mV/V. Conversely, devices with compressive stress SiN_x_ passivation consistently show lower DIBL factors (even as low as 1.6 mV/V). These test data offer compelling evidence that compressive stress-passivated SiN_x_ effectively mitigates the CLM, DIBL, and GIDL effects, thereby suppressing SCEs.

**Figure 5:**
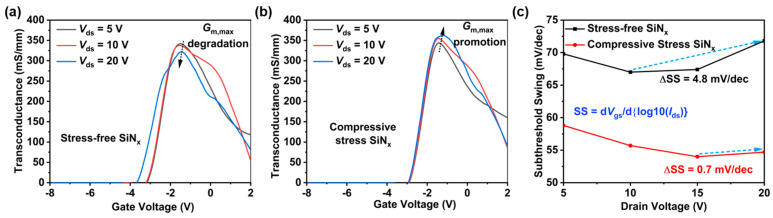
The transconductance at different *V_ds_* biases of devices with (**a**) stress-free SiN_x_ passivation and (**b**) compressive stress SiN_x_ passivation. (**c**) The subthreshold swing at different *V_ds_* biases for each device.

Generally, SCEs lead to a reduction in the gate control capability of the device, resulting in a decreasing trend in transconductance as *V_ds_* increases. The transconductance of both types of devices at different *V_ds_* biases is measured, as shown in Figure 5a,b. As the *V_ds_* bias increases, the devices with stress-free SiN_x_ passivation exhibit a gradual decrease in transconductance, whereas those with compressive stress SiN_x_ passivation demonstrate a gradual increase. The subthreshold swing at different *V_ds_* biases for each device is calculated based on the measured transfer characteristic curves, as depicted in Figure 5c. The calculation formula is as follows:

(2) $$SS=\frac{dV_{gs}}{d\left(\log_{10}I_{ds}\right)}$$

Compared to devices with stress-free SiN_x_ passivation, those with compressive stress SiN_x_ passivation exhibit a lower subthreshold swing (less than 60 mV/dec), with minimal degradation (only increasing by 0.7 mV/dec) as the *V_ds_* bias increases. However, devices with stress-free SiN_x_ passivation experience significant degradation of the subthreshold swing, with an increase of 4.8 mV/V observed from *V_ds_* = 10 V to 20 V. These results further confirm that SiN_x_ stress-engineering techniques are effective in suppressing SCEs.

### 3.3. TCAD Simulation Results and SCE Suppression Mechanism

**Figure 6:**
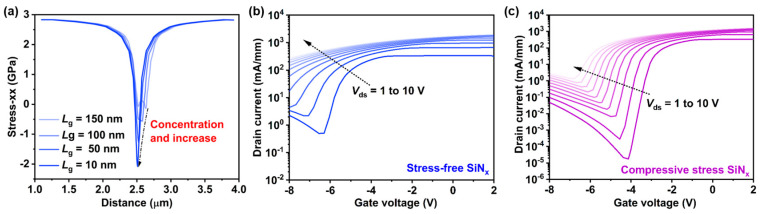
(**a**) Stress distribution for various gate lengths. Transfer characteristic curves at different gate lengths under *V_ds_* = 1 V to 10 V for devices with (**b**) stress-free SiN_x_ and (**c**) compressive stress SiN_x_.

In this study, we established a simulation-based physical model for each GaN RF device using technology computer-aided design (TCAD) Sentaurus, with the model sourced from Silvaco’s Victory Process simulator, specifically focusing on the impact of nitride intrinsic stress. These models were calibrated following the procedure outlined in reference [35], and the transfer and output characteristics of both the experimental and simulation results were accurately fitted in our previous studies [36,37], thereby confirming the model’s accuracy. Based on this model, we investigated the suppression effect of SiN_x_ stress engineering on SCEs as the device size is scaled down to a *L*_g_ of 10 nm. Figure 6a illustrates the stress distribution at the AlGaN/GaN heterojunction for compressive stress SiN_x_ devices with gate lengths ranging from 10 to 150 nm. As expected by the edge force model [38], the stress (compression) field peaks at each gate edge. For the 10 nm gate dimension, because the gate edges get close, the compression at the gate middle piles up and becomes stronger than that of the 150 nm gate. To investigate the effect of SiNx stress engineering on suppressing SCEs in devices with a gate length of 10 nm, we extracted the transfer characteristic curves under *V_ds_* = 1 V to 10 for the devices with stress-free SiN_x_ passivation and compressive stress SiN_x_ passivation. As shown in Figure 6b,c, as the gate length is reduced to 10 nm, the SCEs become increasingly severe. As *V_ds_* increases, the threshold voltage of the device with stress-free SiN_x_ shows a noticeable negative shift, with a DIBL of 840 mV/V. In contrast, the device with compressive stress SiN_x_ passivation exhibits a smaller *V_th_* shift (DIBL of 520 mV/V), confirming that SiN_x_ stress engineering can be effectively applied to suppress SCEs in smaller-sized devices.

**Figure 7:**
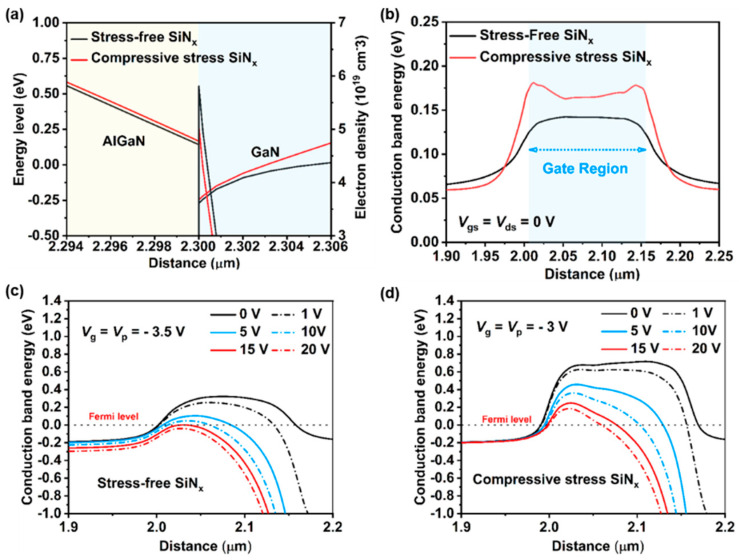
(**a**) Schematic diagrams of the conduction bands (along the y-axis cutline) and (**b**) the conduction band at the heterojunction (along the x-axis cutline) beneath the gate region for each device. Conduction energy band profiles along the channel with different drain bias of those devices (**c**) with stress-free SiN_x_ passivation and (**d**) with compressive stress SiN_x_ passivation.

To comprehensively explore the intrinsic mechanism of the stress-engineered technique in suppressing SCEs, Figure 7a illustrates schematic diagrams of the conduction bands (along the y-axis cutline) beneath the gate region for both types of devices. The introduction of compressive stress SiN_x_ passivation raises the conduction bands of AlGaN and GaN, thereby enhancing channel confinement. Moreover, we extracted the conduction band under *V_ds_* = *V_gs_* = 0 V at the heterojunction (along the x-axis cutline) beneath the gate region, as shown in Figure 7a. Devices with compressive stress SiN_x_ passivation exhibit a 0.05 eV higher conduction band energy compared to those with stress-free SiN_x_ passivation, indicating superior gate control capability. This elevation also accounts for the improved transconductance and subthreshold swing. To explicitly illustrate SCEs in a highly scaled device, we examined the conduction band profiles. Figure 7c,d depict the 1D conduction energy profiles in the channel of each device at pinch-off, calculated at a gate bias of *V*_P_ (pinch-off voltage) with the drain bias varied between 0 and 20 V. The poor aspect ratio results in compromised gate channel control, allowing the drain bias to impact the entire potential barrier within the channel of each device. As shown in Figure 7a, the potential barrier between the source and drain in the GaN HEMTs with stress-free SiN_x_ passivation is lowered as the *V_ds_* bias increases, which directly deteriorates the channel confinement and the output conductance. Furthermore, when the *V_ds_* bias increases above 10 V, the potential barrier between the source and drain decreases to nearly below the quasi-Fermi level. Consequently, the current can flow through the channel without any hindrance. Notably, under the same *V_ds_* bias, devices with compressive stress SiN_x_ passivation maintain a higher potential barrier. Even as *V_ds_* increases to 20 V, the barrier remains higher than the quasi-Fermi level. Therefore, SCEs are greatly improved, maintaining better pinch-off performance and very low output conductance, unlike devices with stress-free SiN_x_ passivation.

### 3.4. Potential Impact on Trapping

**Figure 8:**
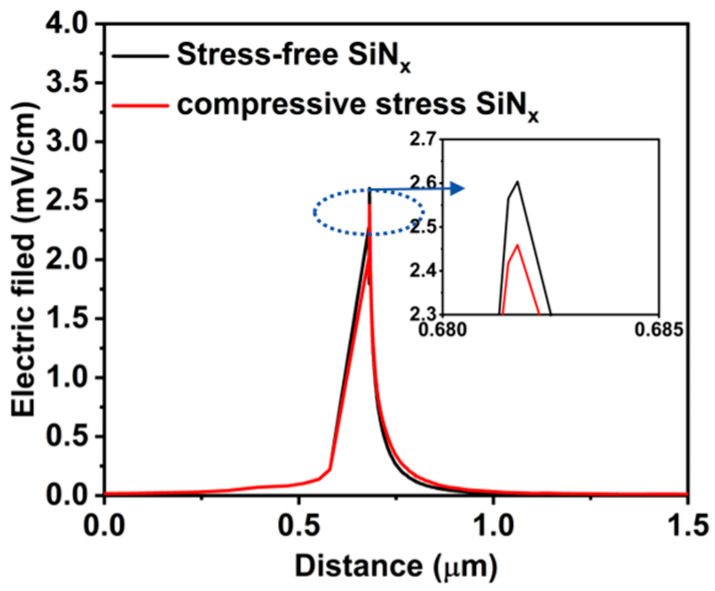
Simulation of electric field under gate region for AlGaN/GaN HEMTs with stress-free SiN_x_ and compressive stress SiN_x_.

In GaN HEMTs, electron trapping can lead to current collapse, knee walk-out, and kink effects, which are critical for the stability of RF applications [39]. This trapping is primarily related to surface and buffer layer defects within the device and is exacerbated under high electric fields [40]. SiN passivation effectively reduces surface defect density, helping to mitigate these adverse effects. As shown in Figure 8, we extracted the peak electric field in the gate region for those devices; the peak electric field in the device with compressive stress SiN_x_ is reduced by 0.15 MV/cm compared to the device with stress-free SiN_x_, which explains the reduction in leakage current and improvement in breakdown voltage shown in Figure 3. Thus, compressive stress SiN_x_ passivation not only further reduces surface defects but also optimizes the electric field distribution, alleviating stress concentration in high-field areas and effectively suppressing defect trapping. This enhancement significantly boosts the device’s dynamic performance and reliability, offering more stable operating conditions for high-performance RF applications.

## 4. Conclusions

In summary, this work delved into the influence of stress engineering on SCEs in GaN HEMTs. Through a comprehensive analysis encompassing DIBL, GIDL, and CLM, it systematically examined the distinctions between the devices with stress-free and compressive stress SiN_x_ passivation. TCAD Sentaurus simulation outcomes revealed that compressive stress SiN_x_ passivation effectively raises the conduction band of AlGaN and GaN beneath the gate region, enhancing channel confinement. Furthermore, it raises the potential barrier at the heterojunction under the gate, especially at the gate edge, maintaining it above the quasi-Fermi level even with *V_ds_* increasing to 20 V. As a result, stress-engineered techniques effectively suppress SCEs, providing a practical and efficient approach for their mitigation in future GaN RF devices. Furthermore, the application of compressively stressed SiN significantly reduces the peak electric field in the gate region, resulting in lower leakage currents and higher breakdown voltages. The reduction in the peak electric field also benefits the minimization of defect trapping, which is expected to suppress current collapse, knee walk-out, and kink effects, thereby achieving high-performance and high-reliability GaN RF devices.

However, the SiN_x_ stress-engineered techniques in GaN HEMTs face several challenges that need to be addressed. One critical issue is achieving uniformity control across large wafers, as precise deposition parameters are essential for ensuring consistent device performance. Additionally, the effects of temperature on device behavior warrant further investigation, particularly since stress relaxation or amplification at extreme temperatures could impact reliability. It is also important to explore the applicability of this technique to other barrier materials, such as AlN or InAlN, and to various device architectures to validate its universality. Effectively tackling these challenges is vital for enhancing the practical implementation and performance optimization of GaN HEMTs in high-power and high-frequency applications.

## Data Availability

The data that support the findings of this study are available from the corresponding authors upon reasonable request.
